# The complete mitochondrial genome of a walnut weevil, *Alcidodes juglans* Chao (Coleoptera: Curculionidae)

**DOI:** 10.1080/23802359.2018.1535854

**Published:** 2018-11-25

**Authors:** Kangkang Xu, Xiaoyulong Chen, Lin Xu, Wenjia Yang, Yawei Wang, Can Li

**Affiliations:** a Guizhou Provincial Key Laboratory for Rare Animal and Economic Insect of the Mountainous Region, College of Biology and Environmental Engineering, Guiyang University, Guiyang, China;; b College of Tobacco Science, Guizhou University, Guiyang, China

**Keywords:** *Alcidodes juglans* Chao, walnut weevil, mitochondrial genome

## Abstract

The walnut weevil, *Alcidodes juglans* Chao (Coleoptera: Curculionidae), is an important agricultural pest and distributed widely in China. The complete mitochondrial genome of *A. juglans* is 15,638 bp long, and consists of 13 protein-coding genes (PCGs), two ribosomal RNA genes, 21 transfer RNA (tRNA) genes and a putative control region (GenBank accession No. MH819192). The *trnI* gene has not been observed in the *A. juglans* mitogenome. The nucleotide composition is significantly biased (A, G, C, and T was 38.35%, 10.02%, 14.96%, and 36.67%, respectively) with A + T contents of 75.02%. All of the 21 tRNAs have the typical cloverleaf structure, with an exception for *trnS_1_*(AGN). All PCGs are initiated by ATN codons, except for *cox1* with AAT instead. Ten PCGs use a common stop codon of TAA or TAG, whereas the remaining three were terminated with a single T. The phylogenetic relationships based on neighbour-joining method showed that *A. juglans* is closely related to *Naupactus xanthographus*, which is in accordance with the traditional morphological classification.

The walnut weevil, *Alcidodes juglans* Chao (Coleoptera: Curculionidae), is an important agricultural pest and distributed widely in China. The larvae of *A. juglans* bore tunnels into the centre of fruits, and spend their larval stage inside host plants. It causes great economic losses in walnut cultivated areas (Liu and Feng 2014).The specimen of *A. juglans* used in this study were collected from Hezhang County, Guizhou Province, China (N27°05′, E104°37′), and deposited in the insect specimen room of Guiyang University with an accession number GYU-Col-20180001.

The complete mitogenome of *A. juglans* is a closed-circular molecule of 15,638 bp in length (GenBank accession No. MH819192), and containing the typical set of 13 protein-coding genes (PCGs), two ribosomal RNA genes (*rrnL* and *rrnS*), 21 transfer RNA genes (tRNAs), and a putative control region. The gene order and organization of *A. juglans* are consistent with those of putative ancestor of insects (Boore 1999). The *trnI* was not found in the *A. juglans* mitogenome, as observed in *Sympiezomias velatus* (Tang et al. 2017) and *C. buqueti* (Yang et al. 2018), two completely sequenced species in Coleoptera. The nucleotide composition of the mitogenome of *A. juglans* is significantly biased (A, G, C, and T was 38.35%, 10.02%, 14.96%, and 36.67%, respectively) with A + T contents of 75.02%. The AT-skew and GC-skew of this genome were 0.022 and −0.330, respectively. Fourteen genes were oriented on the N-strand, whereas the others were transcribed on the J-strand.

The *A. juglans* mitogenome harbours a total of 16 bp overlapping sequences in six regions. The longest overlap is 7 bp in length, and located between *atp8* and *atp6*. This mitogenome has a total of 182 bp intergenic spacer sequences, which is made up of 14 regions in the range from 1 to 67 bp. The largest intergenic spacer sequence of 67 bp is located between *trnS_2_* and *nad1*. The control region was located between *rrnS* and *trnQ* genes with a length of 803 bp, and the A + T content was 83.44%. With an exception for *trnS_1_*(AGN), all tRNAs have the typical cloverleaf structure, which are similar to those reported in most animal mitogenomes (Wolstenholme 1992; Yuan et al. 2016). The length of these tRNAs ranged from 63 bp (*trnT* and *trnE*) to 71 bp (*trnK*), A + T content ranged from 59.42% (*trnR*) to 90.91% (*trnD*). Two rRNAs (*rrnL* and *rrnS*) are located between *trnL_1_* and *trnV*, and between *trnV* and the control region, respectively. The *rrnL* was 1323 bp in length with A + T content of 77.63%, and the *rrnS* was 789 bp in length with A + T content of 73.26%.

The initial codons for 12 PCGs of *A. juglans* were the canonical putative start codons ATN (ATG for *atp6*, *cox3*, *nad4L*, and *cob*; ATT for *nad3*, *nad5*, *nad6*, and *nad2*; ATA for *atp8*, *nad4*, and *nad1*; ATC for *cox2*). However, *cox1* used AAT as start codon, as observed in *Tribolium castanum* (Liu et al. 2014), another completely sequenced species in Coleoptera. The typical termination codon (TAA or TAG) occurs in 10 PCGs, and the remaining PCGs including *cox1*, *cox2*, and *nad5* were terminated with a single T. Based on the concatenated amino acid sequences of 13 PCGs, the neighbour-joining method was used to construct the phylogenetic relationship of *A. juglans* with 14 other bettles. The result showed that *A. juglans* is closely related to *Naupactus xanthographus* (Figure 1), which is in accordance with the traditional morphological classification.

**Figure 1.**
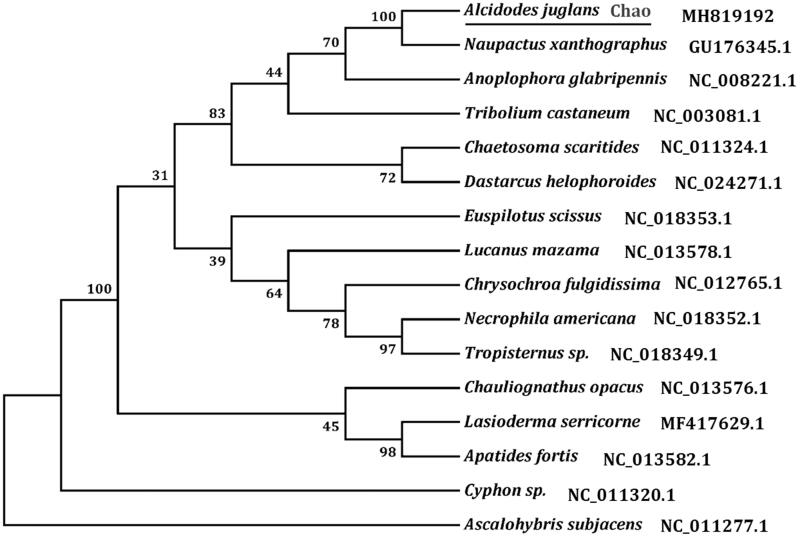
Phylogenetic tree showing the relationship between A. juglans and 14 other beetles based on neighbour-joining method. Ascalohybris subjacens was used as an outgroup. GeneBank accession numbers of each species were listed in the tree.

## References

[CIT0001] Boore JL 1999 Survey and summary: animal mitochondrial genomes. Nucleic Acids Res. 27:1767–1780. 10101183 10.1093/nar/27.8.1767 PMC148383

[CIT0002] Liu CH, Feng LM 2014 Reasons analysis on severer harms by *Alcidodes juglans* Chao. Shanxi Forest Sci Technol. 1:84–86.

[CIT0003] Liu QN, Bian DD, Jiang SH, Li ZX, Ge BM, Xuan FJ, Yang L, Li FC, Zhang DZ, Zhou CL, Tang BP 2014 The complete mitochondrial genome of the red flour beetle, *Tribolium castaneum* (Coleoptera: Tenebrionidae). Mitochondrial DNA. 10:1–3. 10.3109/19401736.2014.953122 25162515

[CIT0004] Wolstenholme DR 1992 Animal mitochondrial DNA: structure and evolution. Int Rev Cytol. 141:173–216. 1452431 10.1016/s0074-7696(08)62066-5

[CIT0005] Tang PA, Zhang L, Li XP, Li FF, Yuan ML 2017 The complete mitogenome of *Sympiezomias velatus* (Coleoptera: Curculionidae). Mitochondrial DNA Part B. 2:449–450. 10.1080/23802359.2017.1357445 PMC7800147 33473858

[CIT0006] Yang WJ, Yang DX, Xu KK, Cao Y, Meng YL, Wu Y, Li GY, Zhang GZ, Wang YW, Li C 2018 Complete mitochondrial genome of the bamboo snout beetle, *Cyrotrachelus buqueti* (Coleoptera: Curculionidae). Mitochondrial DNA Part B. 3:88–89. 10.1080/23802359.2017.1422411 PMC7800430 33474076

[CIT0007] Yuan ML, Zhang QL, Zhang L, Guo ZL, Liu YJ, Shen YY, Shao RF 2016 High-level phylogeny of the Coleoptera inferred with mitochondrial genome sequences. Mol Phylogenet Evol. 104:99–111. 27497607 10.1016/j.ympev.2016.08.002

